# Migraine chronification as an allostatic disorder: a proof-of-concept study

**DOI:** 10.1007/s10072-023-07293-8

**Published:** 2024-01-23

**Authors:** Calogero Calabrò, Eliana Di Tillo, Umberto Pensato, Corrado Zenesini, Valentina Favoni, Camilla Fontana, Sabina Cevoli, Eliana Tossani, Pietro Cortelli, Silvana Grandi, Giulia Pierangeli

**Affiliations:** 1 Department of Biomedical and NeuroMotor Sciences, University of Bologna, Bologna, Italy; 2 Azienda USL di Bologna, Bologna, Italy; 3 Department of Biomedical Sciences, Humanitas University, via Rita Levi Montalcini 4, 20072 Pieve Emanuele, Milan, Italy; 4 IRCCS Humanitas Research Hospital, via Manzoni 56, 20089 Rozzano, Milan, Italy; 5 IRCCS Istituto Delle Scienze Neurologiche Di Bologna, Bellaria Hospital, Via Altura 3, 40139 Bologna, Italy; 6 Department of Medical and Surgical Sciences, University of Bologna, Bologna, Italy; 7 Department of Psychology, University of Bologna, Bologna, Italy

**Keywords:** Chronic migraine, Allostasis, Stress, Pathophysiology, Primary headache, Risk factors

## Abstract

**Objective:**

The underpinning biologics of migraine chronification are not well understood. We aim to investigate the role of the cumulative burden of stress, namely the allostatic load, in migraine chronification.

**Methods:**

This was a cross-sectional study. The allostatic load was measured with a composite multi-system score (BALI: Bologna Allostatic Load Index), evaluating 20 biomarkers representing four physiological systems: immune, metabolic, cardiovascular, and neuroendocrinological systems. BALI score was subdivided into high score and low score based on the distribution in controls. Migraine patients were included and subclassified into low-frequency episodic migraine group (low-EM group), high-frequency episodic migraine group (high-EM group), and chronic migraine group (CM group).

**Results:**

The distribution of BALI high-score increased in parallel with headache attacks monthly frequency: 16% in low-EM group (*n* = 10), 24% in high-EM group (*n* = 12), and 40% in CM group (*n* = 21) (*p* = 0.017). In a multivariable analysis, the odds ratio of having a high-score BALI in CM patients (vs. low-EM patients) was 2.78 (95% CI 1.07–7.22; *p* = 0.036). Individual BALI biomarkers values which were significantly different among migraine subgroups included systolic blood pressure (*p* = 0.018), diastolic blood pressure (*p* < 0.001), and heart rate (*p* = 0.019).

**Conclusion:**

Our study substantiates this emerging concept of migraine chronification as an allostatic disorder.

**Supplementary Information:**

The online version contains supplementary material available at 10.1007/s10072-023-07293-8.

## Background

Migraine is a complex neurological disorder characterized by recurrent disabling attacks that stressful conditions may trigger [1]. These attacks consist of several phases: prodromal symptoms, aura, headache phase with pain variably accompanied by other symptoms, resolution, and recovery (or postdrome) [2]. The underlying pathophysiology of migraine pain and aura has been extensively evaluated, and much evidence points out that the culprit mechanisms are located in the brain [3]. However, there is little information about the actual causes of migraine and why individuals have a different risk of suffering a migraine attack at some time in life under particular circumstances. To clarify this issue, the brain of patients with migraine has been investigated interictally. Significant differences from healthy controls were found, including the abnormally increased cortical excitability to pain [4], light [5], or smell [6]. Other differences relate to abnormalities in responses that should be adaptive but become impaired or maladaptive, such as altered brainstem processing [7]. In addition, associated changes in gray matter volume [8], impaired adaptive cerebral hemodynamic mechanisms [9], habituation deficiency [10], and an imbalance between energetic supply and demand [11] have been described. Thus, migraine should be considered a brain disease and not simply a recurrent acute pain syndrome, and it should also be considered a continuum in the progression to high frequency and chronic daily headache that occur in some patients [12].

The brain is a central organ of stress [13] that determines what is stressful or potentially stressful and initiates behavioral and physiologic responses that could be either adaptive or maladaptive. These brain responses are mediated via the autonomic nervous system and neuroendocrine mechanisms. In this context, allostasis is the ability to protect the body through increased activity of mediators that typically promote adaptation [14], and allostatic load and overload refer to the wear and tear on the systems (including the brain) that typically support adaptation and normal function as a result of repeated stress and/or allostasis. This conceptualization has paved the way to interpret migraine as a model disease of allostatic load [15, 16] and as a genetically determined behavioral response consistent with sickness behavior [17]. However, few studies have tried to support this theory with biological data. In this study, we aim to investigate the role of the cumulative burden of stress in the process of migraine chronification.

### Objectives

The study’s primary aim was to investigate the potential relationship between the allostatic load, measured with a composite multi-system index (BALI: Bologna Allostatic Load Index), and monthly headache frequency. The second aims were: (i) to assess the contribution of the BALI biomarkers to determine the most significant physiological systems; (ii) to investigate the potential relationship between BALI scores and psychological parameters; (iii) to investigate the potential relationship between monthly headache frequency and psychological parameters.

## Methods

### Design, standard protocol approvals, and participation consent

This was a cross-sectional, monocentric study. The STROBE (Strengthening the Reporting of Observational Studies in Epidemiology) guideline was followed [18]. The study was approved by an independent ethics committee or local institutional review board (protocol number: 14112). Written informed consent was obtained from all enrolled patients and controls for study participation and data publication. All procedures were conducted according to the latest version of the Declaration of Helsinki.

### Setting and study population

Patients referred to the tertiary Headache Centre of Bologna, IRCCS Institute of Neurology, Bologna, Italy, between 2017 and 2021 were proposed to participate in the study. Enrolment was carried out one day per week during the study period due to the limited availability of researchers to support recruitment. Inclusion criteria were: (i) diagnosis of episodic and chronic migraine without aura according to the International Classification of Headache Disorders-Third edition (ICHD-3) [19]; (ii) migraine onset before 40 years of age and (iii) age 18–75 years. Patients were further subclassified, based on headache frequency evaluated prospectively with a 3-month headache diary, into low-moderate frequency episodic migraine group (low-EM group) (1–9 headache days per month), high-frequency episodic migraine group (high-EM group) (10–14 headache days per month), and chronic migraine group (CM group) (≥ 15 days per month). In the case of heterogeneity of headache days per month frequency, patients were classified according to their average frequency during the 3-month study period. Controls were selected among headache patients’ companions (first-degree relatives excluded). The inclusion criteria of controls were: (i) absence of any headache or chronic pain disorder and (ii) age 18–75 years. Breastfeeding or pregnant women were excluded from both patients and controls, as well as subjects suffering from headache disorders other than migraine, major cardiovascular or cerebrovascular conditions, and mild cognitive impairment.

### Variables and assessment

We collected demographic and anamnestic data, including headache features. All individuals were evaluated by an expert psychologist and neurologist through several self-rating and interview-based validated questionnaires and tests: a revised version of Diagnostic Criteria for Psychosomatic Research (DCPR) [20] to identify subjects with the psychosomatic syndrome Allostatic Overload, Structured Clinical Interview for DSM-5 to assess DSM-5 diagnosis (SCID-5-CV) [21], Migraine Disability Assessment (MIDAS) grade [22], Mini-Mental State Score (MMSE), and Perceived Stress Scale (PSS) [23]. All individuals were comprehensively assessed with blood and 24-h urinary tests. Additionally, weight, height, hip, and waist diameter were collected, as well as heart rate and arterial blood pressure, measured with an automatic sphygmomanometer (OMRON Healthcare) three times after at least 30 min of lying.

### Allostatic load

The allostatic load was measured with a composite multi-system index (BALI: Bologna Allostatic Load Index), evaluating 20 different biomarkers representing four physiological systems:

1. Inflammation and immune system (serum C reactive protein [CRP], serum interleukin-6 [IL-6], serum fibrinogen),
2. Metabolic system (waist-to-hip-ratio [WHR], body mass index [BMI], serum total cholesterol, serum high-density cholesterol [HDL], serum triglycerides, fasting glucose, serum insulin, serum glycosylated hemoglobin [HbA1C]),
3. Cardiovascular system (systolic blood pressure [SBP], diastolic blood pressure [DBP], heart rate [HR])
4. Neuroendocrinological system (serum dehydroepiandrosterone sulfate [DHEA-S], serum cortisol, and 24 h urinary cortisol, norepinephrine, epinephrine, and dopamine)

Each biomarker was dichotomized into high risk (1 point) and low risk (0 points), where high risk was defined as the highest quintile, compared to age- and sex-adjusted normative value, apart from biomarkers inversely related to health outcomes (Table 1). These values were also adjusted according to individual current therapies: (i) 10 mmHg and 5 mmHg were added to systolic and diastolic blood pressures, respectively (anti-hypertensive medication), (ii) 1% was added to HbA1c values (diabetes medication), (iii) total cholesterol level added by 21.24 mg/dl (statins) or reduced by 4% (diuretics), (iv) HDL increased by 10% (beta-blockers) [24]. The composite score was calculated by summing the dichotomous scores of each biomarker (range: 0–20). BALI score was subdivided into a high score and a low score based on control distribution: a BALI score ≥ 6 was observed in 20% of controls (*n* = 12), corresponding to the value nearest to the higher quintile of the distribution; hence, the high-score BALI was defined based on this cutoff.

**Table 1:** Biomarkers assessed for the Bologna Allostatic Load Index (BALI)

Biomarkers	*Normal range*	Highest quintile (1 point)
Inflammatory and immune system		
• Serum CRP • Serum IL-6 • Serum fibrinogen	< 0.5 mg/dL < 5.9 pg/mL 150–400 mg/dL	≥ 0.375 ≥ 4.425 ≥ 337.5
Metabolic system		
• Waist-to-hip ratio • BMI • Serum total cholesterol • Serum HDL • Serum triglycerides • Serum fasting glucose • Serum insulin • HbA1C	0.8–1 18.5–25 < 200 mg/dL 35–77 mg/dL < 150 mg/dL 60–110 mg/dL 1.9–23 µU/die 20–42 mmol/mol	≥ 0.95 ≥ 23.375 ≥ 150 ≤ 46 ≥ 112.5 ≥ 97.5 ≥ 17.725 ≥ 36.5
Neuroendocrine system		
• 24 h urinary epinephrine • 24 h urinary norepinephrine • 24 h urinary dopamine • Serum DHEAS • Serum cortisol • 24 h urinary cortisol	1.7–22.4 µg/die 12.1–85.5 µg/die < 500 µg/die 70–400 µg/dL 67–226 ng/mL 58–403 µg/dL	≥ 17.225 ≥ 67.15 ≥ 375 ≤ 152.5 ≥ 186.25 ≥ 316.75
Cardiovascular system		
• Systolic blood pressure • Diastolic blood pressure • Heart rate	90–140 mmHg 60–90 mmHg 60–100 bpm	≥ 127.5 ≥ 82.5 ≥ 77

*CRP* C reactive protein, *IL-6* interleukin-6, *BMI* body mass index, *HDL* high-density cholesterol, *HbA1C* glycosylated hemoglobin, *DHEA-S* dehydroepiandrosterone sulfate, *bpm* beats per minute

### Statistical analysis

The statistical analysis was performed with Stata SE 14.2. Continuous variables were checked for normality using the Shapiro–Wilk test and presented as mean ± standard deviation (SD) or median and interquartile range (IQR). Continuous variables were compared between the groups by one-way analysis of variance or the Kruskal–Wallis test, depending on the data distribution, followed by the Bonferroni post hoc analysis for multiple comparisons. Categorical variables were presented as absolute (*n*) and relative frequency (%); they were compared between the groups with the Chi-square test or Fisher’s exact test.

Univariable and multivariable logistic regression models were used to evaluate the association between BALI (dependent variable: high vs. low score), migraine subgroups, and other variables described in the method section (independent variable). In the multivariable model, we added the variables significant in the univariable analysis as covariates. The results were presented as odds ratio (OR) and 95% confidence interval (95% CI). *p* values (two-tailed) < 0.05 were considered significant.

## Results

### Patients, controls, clinical characteristics, and psychiatric disorders prevalence

Among 164 migraine patients included in the study, 37% (*n* = 61) were low-EM, 30% (*n* = 50) were high-EM, and 32% (n = 53) were CM. Epidemiological and behavioral characteristics were similar among the migraine subgroups. Conversely, a significantly increasing trend of psychiatric disorders (major depressive disorder [*p* = 0.050] and generalized anxiety disorders [*p* = 0.038]) was observed (Table 2). A higher MIDAS grade, as expected, was observed among the CM group (*p* < 0.001) and patients with higher migraine frequency, whereas PSS values were similar.

**Table 2:** Demographic and baseline characteristics between migraine subgroups

	Low-EM group	High-EM group	CM group	*p* value
	(*n* = 61)	(*n* = 50)	(*n* = 53)	
**Epidemiological characteristics**				
Age (years), median (IQR)	49 (39–57)	49 (42–55)	50 (43–57)	0.826
Female sex	48 (79%)	38 (76%)	39 (74%)	0.815
**Behavioral characteristics**				
Active smokers	7 (12%)	11 (22%)	12 (23%)	0.220
Heavy alcohol consumers	4 (7%)	3 (6%)	4 (8%)	0.948
Coffee consumers	53 (87%)	42 (84%)	46 (89%)	0.800
Regular exercise activity	27 (45%)	25 (50%)	17 (32%)	0.160
**Psychiatric comorbidities**				
Major depressive disorder	4 (7%)	2 (4%)	9 (17%)	**0.050**
Generalized anxiety disorder	8 (13%)	9 (18%)	17 (32%)	**0.038**
DCPR allostatic overload	18 (30%)	16 (32%)	18 (34%)	0.877
**Disability and stress-related scores**				
MIDAS grade, median (IQR)	2 (1–4)	4 (3–4)	4 (3–4)	** < 0.001**^**a**^
PSS, median (IQR)	20 (13–25)	19 (13–24)	17 (13–23)	0.708

*DCPR* Diagnostic Criteria for Psychosomatic Research, *MIDAS* migraine disability assessment score, *PSS* perceived stress score

^a^*p* < 0.05 between low-EM and high-EM groups and between low-EM and CM groups

Sixty-one controls were included in the study. The median age was 47 (Kruskal–Wallis test = 0.92 vs. patient groups), and 67% (*n* = 41) were females(Chi-square test = 0.52 vs. patient groups). Psychiatric comorbidities were: major depressive disorder (0%), generalized anxiety disorder (5%), while 13% had DCPR syndrome Allostatic Overload. Epidemiological and behavioral characteristics, individual BALI biomarkers scores and values, as well as distribution of BALI composite score of the control group, are illustrated in eTables 1, 2, and 3 and eFig. 1.

### BALI assessment and relationship with psychiatric disorders

**Fig. 1:**
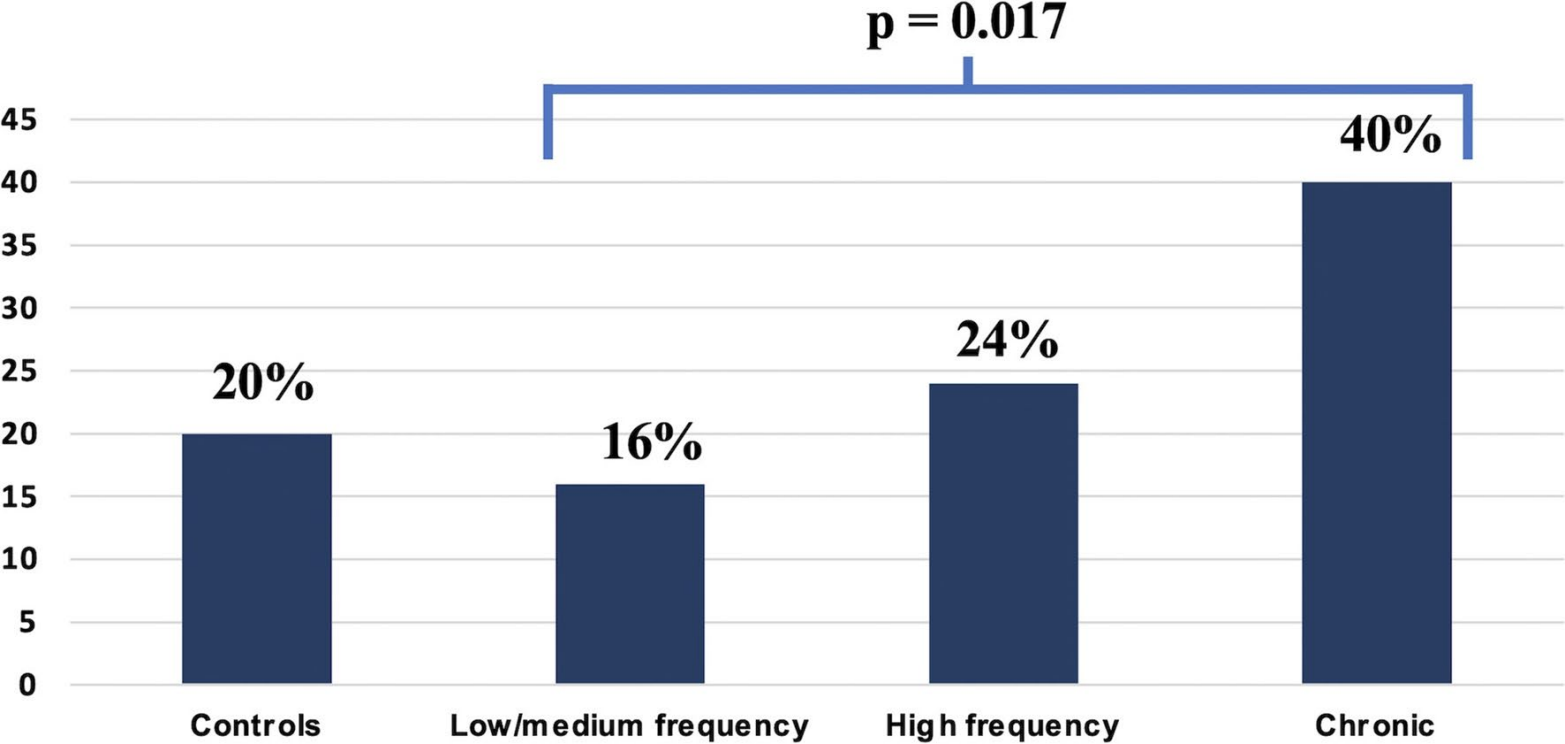
Percentage distribution of BALI (Bologna Allostatic Load Index) high-score among groups. The distribution of BALI high-score increases in parallel with migraine attacks monthly frequency

The distribution of BALI high-score increased in parallel with migraine attacks monthly frequency: 16% in low-EM group (*n* = 10), 24% in high-EM group (*n* = 12), and 40% in CM group (*n* = 21) (*p* = 0.017) (Fig. 1). Among the demographic and clinical characteristics, age, sex, and generalized anxiety disorders had a significant OR of having a high-score BALI. Neither MIDAS, PSS, DCPR diagnosis of Allostatic Overload nor major depressive disorder correlated statistically with BALI. In a univariable analysis, CM (vs. low-EM) patients’ odds ratio to have a high-score BALI was 3.35 (95% C.I: 1.40–8.01, *p* = 0.007).

In a multivariable analysis, adjusted for age, sex, and generalized anxiety disorder, the OR to have a high-score BALI in CM (vs. low-EM) patients was 2.78 (95% C.I.: 1.07–7.22, *p* = 0.036) (Table 3).

**Table 3:** Multivariate analysis adjusted for age, sex, and generalized anxiety disorder

High vs low BALI score	OR	95% CI	*p* value
Low-EM group	*ref*	*ref*	*ref*
High-EM group	1.61	0.59 – 4.40	0.349
CM group	2.78	1.07 – 7.22	**0.036**
*Potential confounding variables*			
Age Sex Generalized anxiety disorder	1.05 2.93 3.74	1.02 – 1.10 1.22 – 7.06 1.49 – 9.34	**0.007** **0.016** **0.005**

*OR* odds ratio, *CI* confidence interval

### Individual BALI parameters distribution

Individual BALI biomarkers values, which were significantly different among migraine subgroups, included systolic blood pressure (*p* = 0.018), diastolic blood pressure (*p* < 0.001), and heart rate (*p* = 0.019) (Table 4). The distribution of BALI biomarkers scores among migraine subgroups is shown in eTable 4.

**Table 4:** Distribution of BALI biomarkers values among groups

Biomarkers	Low-EM Median (IQR)	High-EM Median (IQR)	CM Median (IQR)	p-value
Inflammatory and immune system				
• Serum CRP (mg/dL) • Serum IL-6 (pg/mL) • Serum fibrinogen (mg/dL)	0.10 (0.05–0.15) 2.5 (2.0–3.6) 283 (239–309)	0.12 (0.05–0.24) 2.0 (1.8–3.5) 276 (252–307)	0.08 (0.006–0.23) 2.2 (2.4–4.0) 286 (237–334)	0.531 0.152 0.689
Metabolic system				
• Waist-to-hip ratio • BMI • Serum total cholesterol (mg/dL) • Serum HDL (mg/dL) • Serum triglycerides (mg(dL) • Serum fasting glucose (mg/dL) • Serum insulin (µU/die) • HbA1C (mmol/mol)	0.83 (0.78–0.89) 23.9 (22–25.2) 193 (169–210) 54 (47–62) 79 (59–103) 84 (80–91) 4.2 (2.9–5.9) 34 (32–36)	0.84 (0.79–0.88) 25 (22.5–27) 195 (176–207) 55 (45–63) 78 (62–98) 85 (80–92) 4.4 (3.1–7.1) 34 (31–37)	0.86 (0.80–0.91) 23.6 (21.8–26.1) 190 (161–218) 52 (46–59) 82 (62–105) 86 (81–91) 4.6 (3.0–7.1) 35 (33–36)	0.621 0.562 0.887 0.521 0.744 0.963 0.831 0.231
Neuroendocrine system				
• 24 h urinary epinephrine (µU/die) • 24 h urinary norepinephrine (µU/die) • 24 h urinary dopamine (µU/die) • Serum DHEAS (µU/dL) • Serum cortisol (ng/mL) • 24 h urinary cortisol (µU/dL)	6.5 (3.2–12) 41.8 (28.6–51.6) 220 (170–270) 85 (46–135) 91 (73–106) 113 (85–154)	6.5 (3.9–11.5) 44.0 (30.0–54.5) 235 (185–281) 94 (54–148) 90 (78–116) 129 (98–180)	6.8 (4.3–12.2) 39.0 (30–52.6) 219 (178–273) 71 (44–127) 96 (77–120) 120 (88–176)	0.660 0.706 0.883 0.543 0.590 0.317
Cardiovascular system				
• Systolic blood pressure (mmHg) • Diastolic blood pressure (mmHg) • Heart rate (bpm)	102 (92–110) 70 (65–73) 64 (60–69)	110 (97–120) 75 (68–82) 67 (62–75)	105 (97–115) 75 (68–81) 68 (65–73)	**0.018**^**a**^ ** < 0.001**^**a, b**^ **0.019**^**a**^

*CRP* C reactive protein, *IL-6* interleukin-6, *BMI* body mass index, *HDL* high-density cholesterol, *HbA1C* glycosylated hemoglobin, *DHEA-S* dehydroepiandrosterone sulfate, *IQR* interquartile range, *bpm* beats per minute

^a^*p* < 0.05 between low-EM and high-EM groups

^b^*p* < 0.05 between low-EM and CM groups

## Discussion

The biology of migraine chronification is not well understood, and several risk factors have been identified, including female sex, higher baseline migraine attacks frequency, depression, cutaneous allodynia, and medication overuse [25].

Based on the possibility that repeated and chronic stressors may be associated with maladaptive remodeling of brain networks, including prefrontal, hippocampal, and amygdala circuits, putatively leading to migraine chronification [16, 17], we explored the potential contributing role of an increasing allostatic load in the process of migraine chronification. Notably, we found a direct relationship between the BALI score and the migraine disease burden. Accordingly, a progressively increased risk of having a high-score BALI was observed in high frequency (OR = 1.61, 95% CI 0.59–4.40) and chronic (OR = 2.78, 95% CI 1.07–7.22) migraine groups compared to low-frequency episodic migraine patients. These results align with previous theoretical models proposing that structural and functional brain changes related to increasing migraine attack frequency may reflect a proportional escalation of the allostatic load [16, 26].

Regarding the relationship between BALI and psychiatric disorders, generalized anxiety disorder was significantly associated with high-score BALI (OR = 3.74, 95% CI 1.49–9.34). In contrast, other psychiatric disorders, including depression, were more frequent in chronic migraine patients but did not correlate with the BALI score. A well-known bidirectional relationship exists between psychiatric comorbidities and migraine, especially chronic migraine [27, 28]. Our finding may justify a direct causative role of generalized anxiety disorder in migraine chronification, whereas other psychiatric disorders, including depression, may have a more marginal causative role. The psychosomatic diagnosis of allostatic overload, defined according to DCPR criteria [20], as well as PSS and MIDAS, did not correlate with a high-score BALI. These results may indicate that the BALI score is an independent and complementary measure of allostatic load compared to perceived stress and disability scales. Hence, an integrative approach combining biological (allostatic load index) and psychosomatic (self-administered and interview-based questionnaires) parameters may lead to a more tailored classification and possibly treatment for chronic migraine patients. Alternatively, the poor correlation between BALI score and psychosomatic diagnosis of allostatic overload  might reflect the limitations of our allostatic load index to capture the full allostatic burden.

Cardiovascular parameters were the most contributing biomarkers to enhancing our study’s different BALI scores among migraine subgroups. This could be explained by the hypertension-related disruption of the endothelial function and cerebral blood flow that might affect the trigemino-vascular system [29, 30]. Due to these biological changes, the duration and frequency of migraine attacks may increase, leading to migraine chronification [30]. Accordingly, arterial hypertension is one of the most critical risk factors for migraine chronification [31, 32]. A direct association between migraine frequency and the risk of developing cardiovascular events has also been observed [31, 32]. Nonetheless, a contributing role of cardiovascular adverse events related to analgesic overuse in high-frequency and chronic migraine patients cannot be excluded from our cohort.

Our study also proposed a novel allostatic load index, namely the BALI score.

Several different allostatic load indexes have been previously proposed in the literature, consistently reporting a direct relationship with mortality and morbidity outcomes, including obesity, cardiovascular diseases, arthritis, and diabetes, as well as health outcomes [33–36]. The revision of these studies highlighted the importance of evaluating concomitantly multiple biomarkers reflecting multiple systems since no single biological parameter reliably reflects the allostatic load. Unfortunately, there is no validated gold standard among the different composite allostatic load indexes proposed so far, limiting the interpretations and comparability of the studies. Nonetheless, few pivotal requisites have been broadly recognized, such as evaluating at least eight biomarkers representing the immune, metabolic, cardiovascular, and neuroendocrine systems that should be adjusted based on the potential effects of pharmacological therapies [37, 38]. Our composite score has the advantage of concomitantly evaluating 20 biomarkers, reflecting all the systems above, and we validated it on a group of controls with no history of headache. Only one previous study assessed the allostatic load in migraine, yet it included patients based on self-administered questionnaires and confronted the index among perimenopausal migraine patients and controls with no stratification based on the disease burden [39]. Since external and internal stressors, namely the allostatic load, arguably influence the migraine burden and not the migraine incidence, we decided to assess the BALI score only among migraine patients, excluding controls in the analysis, to define its truly contributing role in aggravating the disease burden.

Some potential limitations of the current study require an in-depth discussion.

Considering the cross-sectional nature of our study, the causal-effect relationship remains uncertain. Indeed, we cannot exclude that the increase of allostatic load merely reflects a high frequency/chronic headache. However, the well-known pathophysiological mechanisms of the migraine attack as a response to a stressor arguably suggest that chronic stressors may lead to chronic headache. Nonetheless, a vicious circle where these two variables are reciprocally influenced might also be considered.

The selection of patients’ companions as the control group might have introduced some biases since they share environmental and behavioural features with the patients.

Another potential limitation of our study is that migraine subgroups were defined based on headache frequency, irrespective of migraine treatment history. Hence, our study possibly considered chronic/high-frequency patients who have previously responded to preventive medications as the low-migraine group. However, patients in the chronic-migraine subgroup were likely also refractory to preventive treatment; therefore, they genuinely reflected a severe migraine burden. Additionally, the heterogeneity of migraine treatments during the study period in our cohort was another potential confounder. Finally, as all the previous allostatic load scores proposed, the BALI score has not been validated; thus, future studies will need to assess its validity.

## Conclusions

Our study revealed a potential pathogenic role of allostatic load in migraine chronification, corroborating the concept of chronic migraine as a maladaptive stress response of a susceptible threatened brain. Future prospective and more extensive studies are warranted to confirm our results.

### Supplementary Information

Below is the link to the electronic supplementary material.

Supplementary file1 (DOCX 38 KB)

## Data Availability

The corresponding author takes full responsibility for the data, the analyses and interpretation, and the conduct of the research. She has full access to all of the data and the right to publish any data separate and apart from any sponsor. The datasets generated during and/or analyzed during the current study are available from the corresponding author on reasonable request.
