# Supplementary material for: Migraine chronification as an allostatic disorder: a proof-of-concept study
Source: Neurol Sci. 2024 Jan 23;45(6):2775–82. doi: 10.1007/s10072-023-07293-8 (PMC11081979; doi:10.1007/s10072-023-07293-8)
Supplement: Supplementary file 1 — Supplementary file1 (DOCX 38 KB) [file 10072_2023_7293_MOESM1_ESM.docx]

**Supplementary Table 1. Demographic and clinical characteristics of controls**

|  | | **Controls** |  |
| --- | --- | --- | --- |
|  | | **(n=61)** |  |
| **Demographic characteristics** | |  |  |
| Age (years), median (IQR) | | 47 (35-58) |  |
| Female sex | | 41 (67%) |  |
| **Behavioral characteristics** | |  |  |
| Active smokers | | 18 (30%) |  |
| Heavy alcohol consumers | | 8 (13%) |  |
| Coffee consumers | | 55 (90%) |  |
| Regular exercise activity | | 43 (60%) |  |
| **Psychiatric comorbidities** |  | | |
| Major depressive disorder | | 0 (0%) |  |
| Generalized anxious disorder | | 3 (5%) |  |
| DCPR Allostatic Overload | | 8 (13%) |  |
| **Disability and stress related scores** | |  |  |
| PSS, median (IQR) | | 14 (12-19) |  |

DCPR: Diagnostic Criteria for Psychosomatic Research; PSS: perceived stress score.

| **Biomarkers** | ***Median (IQR)*** |
| --- | --- |
| Inflammatory and immune system   - Serum CRP (mg/dL) - Serum IL-6 (pg/mL) - Serum fibrinogen (mg/dL) | 0.09 (0.05-0.16)  2.0 (2.0-3.6)  277 (232-307) |
| Metabolic system   - Waist-to-hip-ratio - BMI - Serum total cholesterol (mg/dL) - Serum HDL (mg/dL) - Serum triglycerides (mg(dL) - Serum fasting glucose (mg/dL) - Serum insulin (µU/die) - HbA1C (mmol/mol) | 0.84 (0.78-0.90)  24.0 (21.0-26.3)  192 (160-215)  51 (43-63)  77 (58-108)  87 (82-92)  3.9 (3.1-5.8)  34 (32-36) |
| Neuroendocrine system   - 24h urinary epinephrine (µU/die) - 24h urinary norepinephrine (µU/die) - 24h urinary dopamine (µU/die) - Serum DHEAS (µU/dL) - Serum cortisol (ng/mL) - 24h urinary cortisol (µU/dL) | 7.0 (4.3-12.3)  39.1 (32.2-47.6)  231 (190-309)  109 (58-186)  97.5 (79.0-114.5)  130 (99-189) |
| Cardiovascular system   - Systolic blood pressure (mmHg) - Diastolic blood pressure (mmHg) - Heart rate (ppm) | 103 (97-113)  70 (62-78)  64 (59-69) |

**Supplementary Table 2. Distribution of BALI biomarkers values among controls**

CRP: C reactive protein; IL-6: interleukin-6; BMI: body max index; HDL: high-density cholesterol; HbA1C: glycosylated hemoglobin; DHEA-S: Dehydroepiandrosterone sulfate; IQR: interquartile range.

**Supplementary Table 3. Distribution of BALI biomarkers scores among controls**

| **Biomarkers** | ***n (%)*** |
| --- | --- |
| Inflammatory and immune system   - Serum CRP - Serum IL-6 - Serum fibrinogen | 6 (10%)  13 (21%)  9 (15%) |
| Metabolic system   - Waist-to-hip-ratio - BMI - Serum total cholesterol - Serum HDL - Serum triglycerides - Serum fasting glucose - Serum insulin - HbA1C | 7 (11%)  32 (52%)  48 (83%)  18 (30%)  15 (25%)  8 (13%)  1 (2%)  13 (21%) |
| Neuroendocrine system   - 24h urinary epinephrine - 24h urinary norepinephrine - 24h urinary dopamine - Serum DHEAS - Serum cortisol - 24h urinary cortisol | 3 (5%)  3 (5%)  9 (15%)  38 (62%)  1 (2%)  1 (2%) |
| Cardiovascular system   - Systolic blood pressure - Diastolic blood pressure - Heart rate | 3 (5%) 5 (8%)  4 (7%) |

CRP: C reactive protein; IL-6: interleukin-6; BMI: body max index; HDL: high-density cholesterol; HbA1C: glycosylated hemoglobin; DHEA-S: Dehydroepiandrosterone sulfate.

**Supplementary Table 4. Distribution of BALI biomarkers scores among migraine subgroups**

| **Biomarkers** | **Low-EM**  **n (%)** | **High-EM**  **n (%)** | **CM**  **n (%)** | **p-value** |
| --- | --- | --- | --- | --- |
| Inflammatory and immune system   - Serum CRP - Serum IL-6 - Serum fibrinogen | 1 (2%)  10 (16%)  8 (13%) | 2 (4%)  11 (23%)  7 (14%) | 8 (15%)  10 (19%)  12 (23%) | **0.011**  0.725  0.349 |
| Metabolic system   - WHR - BMI - Serum total cholesterol - Serum HDL - Serum triglycerides - Serum fasting glucose - Serum insulin - HbA1C | 5 (8%)  36 (59%)  53 (91%)  15 (25%)  13 (21%)  6 (10%)  2 (3%)  11 (18%) | 7 (14%)  31 (62%)  47 (96%)  14 (28%)  9 (18%)  5 (10%)  0 (0%)  13 (26%) | 6 (11%)  27 (51%)  42 (82%)  15 (28%)  12 (23%)  5 (9%)  2 (4%)  13 (25%) | 0.638  0.497  0.071  0.883  0.836  0.995  0.401  0.557 |
| Neuroendocrine system   - 24h urinary epinephrine - 24h urinary norepinephrine - 24h urinary dopamine - Serum DHEAS - Serum cortisol - 24h urinary cortisol | 9 (15%)  9 (15%)  3 (5%)  49 (80%)  2 (3%)  0 (0%) | 4 (9%)  5 (10%)  2 (4%)  39 (78%)  2 (4%)  1 (2%) | 10 (20%)  5 (10%)  5 (10%)  42 (79%)  2 (4%)  4 (8%) | 0.295  0.674  0.439  0.956  0.978  0.055 |
| Cardiovascular system   - SBP - DBP - HR | 2 (3%)  3 (5%)  6 (10%) | 2 (4%)  12 (24%)  10 (20%) | 7 (13%)  12 (23%)  7 (13%) | 0.070  **0.009**  0.302 |

CRP: C reactive protein; IL-6: interleukin-6; WHR: Waist-to-hip-ratio; BMI: body max index; HDL: high-density cholesterol; HbA1C: glycosylated hemoglobin; DHEA-S: Dehydroepiandrosterone sulfate; SBP: systolic blood pressure; DBP: diastolic blood pressure; HR: heart rate

**Supplementary Figure 1. Cumulative percentage distribution (absolute numbers) of BALI scores in controls are presented as histograms.** Note that a BALI score ≥ 6 was observed in 20% of controls (n=12), corresponding to the value nearest to the higher quintile of the distribution.
